# Atypical visual processing in a mouse model of autism

**DOI:** 10.1038/s41598-020-68589-9

**Published:** 2020-07-24

**Authors:** Ning Cheng, Eden Pagtalunan, Abdulrahman Abushaibah, Jessica Naidu, William K. Stell, Jong M. Rho, Yves Sauvé

**Affiliations:** 1 Alberta Children’s Hospital Research Institute (ACHRI), Cumming School of Medicine, University of Calgary, Calgary, AB T2N 4N1 Canada; 2 O’Brien Centre for the Bachelor of Health Sciences, Cumming School of Medicine, University of Calgary, Calgary, AB Canada; 3 Department of Cell Biology and Anatomy, Alberta Children’s Hospital Research Institute and Hotchkiss Brain Institute, Cumming School of Medicine, University of Calgary, Calgary, AB Canada; 4 Department of Ophthalmology and Visual Sciences, University of Alberta, Edmonton, AB Canada; 5 Department of Physiology, University of Alberta, Edmonton, AB Canada; 6 Present Address: Departments of Neurosciences and Pediatrics, University of California San Diego, Rady Children’s Hospital, San Diego, CA USA

**Keywords:** Autism spectrum disorders, Visual system

## Abstract

Human social cognition relies heavily on the processing of various visual cues, such as eye contact and facial expressions. Atypical visual perception and integration have been recognized as key phenotypes in individuals diagnosed with autism spectrum disorder (ASD), and may potentially contribute to impediments in normal social development, a hallmark of ASD. Meanwhile, increasing studies on visual function in ASD have pointed to detail-oriented perception, which has been hypothesized to result from heightened response to information of high spatial frequency. However, mixed results of human studies have led to much debate, and investigations using animal models have been limited. Here, using BTBR mice as a model of idiopathic ASD, we assessed retinal stimulus processing by full-field electroretinogram and found impaired photoreceptor function and retina-based alterations mostly in the cone pathway. Using the optokinetic reflex to evaluate visual function, we observed robustly enhanced visual response to finer spatial details and more subtle contrasts at only higher spatial frequencies in the BTBR mice, under both photopic and scotopic conditions. These behavioral results, which are similar to findings in a subset of ASD patients, indicate a bias toward processing information of high spatial frequencies. Together, these findings also suggest that, while enhancement of visual behaviors under both photopic and scotopic conditions might be due to alterations in visual processing common to both rod and cone pathways, these mechanisms are probably downstream of photoreceptor function.

## Introduction

Autism spectrum disorder (ASD) is an increasingly prevalent neurodevelopmental disorder, characterised by deficits in social interactions and by restrictive behaviours and interests^1–4^. Although many genetic changes have been linked to ASD, the causes of the vast majority of cases remain unknown*.* Clues might be found in altered processing of sensory inputs, which are required for a comprehensive and accurate interpretation of the social environment. Effective sensory processing is essential to higher-level cognition, as behavioural responses rely on processing and integration of sensory stimuli. Changes at any level of sensory processing pathways might result in changes of behaviour, with potentially debilitating effects on social competence^5^. Atypical processing of sensory stimuli has been recognized recently as a potentially significant cause of ASD symptoms, and inefficient sensory integration, as well as over- or under-responsiveness to specific environmental sensory triggers, have been abundantly reported in ASD studies^6,7^. Such behavioural indicators include extreme sensitivity to certain auditory signals or textures and to smell, as well as altered pain thresholds.

Vision plays a vital role in the accurate interpretation of social conditions, and is a determinant of certain behavioural responses. Atypical visual processing can cause an individual to miss important social cues, such as eye contact and nuances in facial expression or skin pigmentation, and ultimately can have deleterious effects on the learning and acquisition of social skills^8–11^. Clinically, atypical visual phenotypes are common in ASD patients^12–14^, and many studies have reported a preference towards intricate details of a visual scene (“local details”) in ASD, as opposed to a contextual understanding of the image as a whole (“global structure”). It has been hypothesized that these phenotypes might be due to alterations in neural processing of spatial vision, or to a relatively increased response to information of high spatial frequencies^8–11^. However, there have been mixed results from studies in patients, and much debate continues. Establishing a reliable animal model for studying ASD-related alterations in visual processing might reveal the nature and underlying mechanisms of altered visual function observed in these patients. However, such studies in animal models of ASD have been limited^15–20^.

The BTBR T + tf/J (BTBR) inbred mouse strain is a model of idiopathic ASD, exhibiting robust core behavioural symptoms—specifically, deficient communication and sociability, and excessive repetitive behaviours^21–25^. In addition, BTBR mice display neuroanatomical features relevant to ASD, such as agenesis of the corpus callosum^21^. Human studies have suggested that dysgenesis of the corpus callosum could be a major risk factor for developing autism^26^, and neuroimaging findings have consistently revealed atypical developmental trajectory of the size and microstructure of the corpus callosum in the patients^27,28^. Therefore, whether atypical visual processing also occurs in this model warrants further investigation. We previously reported that retinofugal projections are abnormal in BTBR mice, suggesting a potential role for alterations in post-retinal visual pathways^29^. The present study sought to further define visual phenotypes in this model. We used recording of the electroretinogram (ERG) to probe retinal function^30,31^, and optokinetic responses (OKR) to assess not only retinal, but also in part post-retinal processing^32–35^. Both tests were performed under both scotopic (rod-dominated) and photopic (cone-dominated) conditions, in order to probe preferentially rod- versus cone-driven pathways. The results were compared with those from C57BL/6J (B6) mice, an inbred strain with normal levels of social activity and repetitive behaviors, and most often used as the control for the BTBR mice in studies related to autism^21^. Our findings point to retina-based alterations mostly in the cone pathway, and robustly enhanced visual detection of finer spatial details and more subtle contrasts at higher spatial frequencies, under both scotopic and photopic conditions.

## Methods

### Animal husbandry

Adult male and female B6 and BTBR mice, approximately 8–14 weeks old, were used. Adult mice were chosen, to ensure that retino-geniculo-striate pathway maturation had been reached^29,36^. Breeding pairs for both strains were obtained from the Jackson Laboratory (Bar Harbor, ME, USA) and maintained at the mouse facility of the Cumming School of Medicine at the University of Calgary. Mice were housed in conventional mouse cages, with access to standard mouse chow and water ad libitum, in a humidity- and temperature-controlled room with a 12-h light/dark cycle (light-onset at 07:00). All procedures were performed according to the recommendations of the Canadian Council for Animal Care. The protocol of this study was approved by the Health Sciences Animal Care Committee of the University of Calgary.

### The electroretinogram (ERG) as a measure of retinal processing

**Figure 1:**
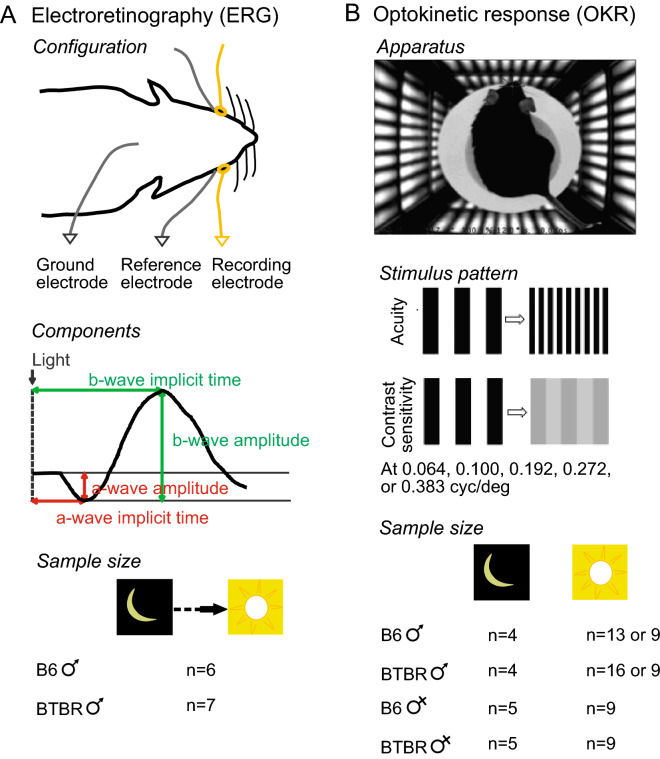
Experimental outline. (**A**) Schematic of recording configuration, measured components of the response, and sample size for ERG. (**B**) Test chamber, stimulus patterns used for acuity and contrast sensitivity tests, and sample size for OKR.

Retinal function was studied electrophysiologically using the full field ERG, following established methods^31,37^. In brief, male BTBR (n = 7) and control B6 (n = 6) mice were dark-adapted overnight and prepared under dim red light for ERG recordings (Fig. 1A). Following anesthesia with a mixture of ketamine (62.5 mg/kg i.p.) and xylazine (12.5 mg/kg i.p.) and pupillary dilation with 1% tropicamide, animals were placed on a homeothermic blanket to maintain their body temperature at 38 °C. Simultaneous bilateral recording was achieved with active gold loop electrodes (placed on each cornea) and subdermal platinum reference electrodes (placed behind each eye); a subdermal platinum ground electrode was placed on the scruff of the neck. Light-stimulation (10 µs flashes), signal amplification (0.3–300 Hz bandpass), and data acquisition were provided by the Espion E^2^ system (Diagnosys LLC, Littleton, MA, USA). Scotopic testing consisted of single-flash presentations at 19 increasing flash strengths, from − 5.22 to 2.86 log cd∙s m^−2^. Ten minutes after transition from dark- to light- adaptation (30 cd m^−2^ white light background), responses under photopic conditions (30 cd m^−2^ background light) were recorded at 11 increasing flash strengths, ranging from − 1.6 to 2.9 log cd∙s m^−2^ (Fig. 1A).

### The optokinetic reflex (OKR) as a measure of visual function

The optokinetic response (OKR)—an innate visuo-motor reflex—was used to measure spatial contrast sensitivity and spatial acuity behaviourally under both photopic and scotopic conditions^32,33,35^. The OKR, as employed here, is also known as the optocollic response—the characteristic smooth movement of the head and neck in the direction of a moving global visual stimulus, followed by a fast saccadic reset. Spatial acuity is a parameter that represents the ability to detect fine spatial details, whereas spatial contrast sensitivity represents the ability to detect differences in light intensity between adjacent image features. A digital OKR apparatus, as described by Prusky et al.^32^, was utilized in this study to assess visual function. The visual pathway for OKR utilizes the accessory optic system, in which retinal afferents (direction-selective ganglion cells) project to the midbrain terminal nuclei, and signals are relayed to the motor system for the appropriate following movement of the head and neck. In mice, it represents primarily the function of inner-retinal circuitry, with potential influences from cerebral cortex and cerebellar nuclei^38–43^.

Rotating vertical sinusoidal gratings, generated by OptoMotry^®^ (Cerebral Mechanics, Lethbridge, AB, Canada), were displayed on four LCD monitors (Model 1703FP, Dell, Phoenix, AZ, USA) forming the walls of a square chamber, modulated in software to generate a virtual cylinder of uniform, alternating dark and light vertical stripes (Fig. 1B). The spatial frequency and contrast of these gratings were manipulated independently, while keeping the horizontal drift velocity constant. In total, we tested the following B6 and BTBR mice: (1) 4 males and 5 females of each strain, under scotopic conditions (Fig. 1B); and (2) 9–16 males and 9 females of each strain, under photopic conditions (Fig. 1B). Mice were dark-adapted for at least 3 h prior to scotopic testing, and care was taken to ensure continued dark-adaptation during the test. Initially, two experimenters performed the tests using different cohorts of mice, under photopic conditions, resulting in larger sample sizes. As the results were consistent, sample sizes were then reduced.

Under both photopic and scotopic conditions, spatial acuity and contrast sensitivities were assessed using the “four-of-five” criterion, in which the mouse must track movement of the grating four out of five times before a head movement could be accepted as an OKR^33^. To measure spatial acuity, the spatial frequency of the grating stimulus at 100% contrast—starting at 0.100 cyc/deg (cycles per degree)—was increased until an OKR could no longer be elicited. To quantify contrast sensitivity, contrast of the gratings was reduced gradually at specific spatial frequencies (0.064, 0.100, 0.192, 0.272 and 0.383 cyc/deg) until the OKR was absent (Fig. 1B). “Contrast sensitivity” was defined as the reciprocal of the lowest contrast at which the OKR was elicited (100/threshold %contrast).

Under the photopic condition, at mean luminance = 1.98 log cd m^−2^, a FireWire iSight camera (Apple Computer Corp., Mountain View, CA, USA) was used for observing the animal’s behaviour from above. Under the scotopic condition, at mean luminance = − 4.32 log cd m^−2^, a CMOS night-vision camera (Model CM900, Clover Electronics, Cerritos, CA, USA) was used. During scotopic testing, the raised platform on which the mouse was placed was surrounded with a clear cylinder (inner diameter of 19.5 cm, 0.8 cm thick) lined with 7 neutral density (ND) filters (Lee Filters, Toronto, ON, Canada), ND = 0.9 each, for light-attenuation. The clear cylinder itself had no measurable effect on visual function^33^. The floor of the OKR chamber, as well as the bottom and top rims of the cylinder, were lined with black cloth to ensure that the dark-adapted state of the mouse was not compromised by light leaking around the filter assembly.

### Statistical analyses

For ERG intensity/response functions, data from B6 and BTBR mice were compared using two way repeated measures ANOVA, followed (if P < 0.05) by Sidak post-hoc pairwise comparison (Graph-Pad Software Inc., San Diego, CA, USA). For ERG dot plots, the data were compared using the non-parametric Mann–Whitney U test (SPSS Statistics, Version 25, IBM Corp, Armonk, NY, USA). For OKR data, acuity and contrast sensitivity at each spatial frequency of B6 and BTBR mice, under scotopic or photopic conditions, were compared using the Mann–Whitney U test. Figures display the means of group data, and error bars indicate the standard errors of the means (SEM).

## Results

### Under scotopic conditions, BTBR mice displayed decreased a-wave amplitudes

**Figure 2:**
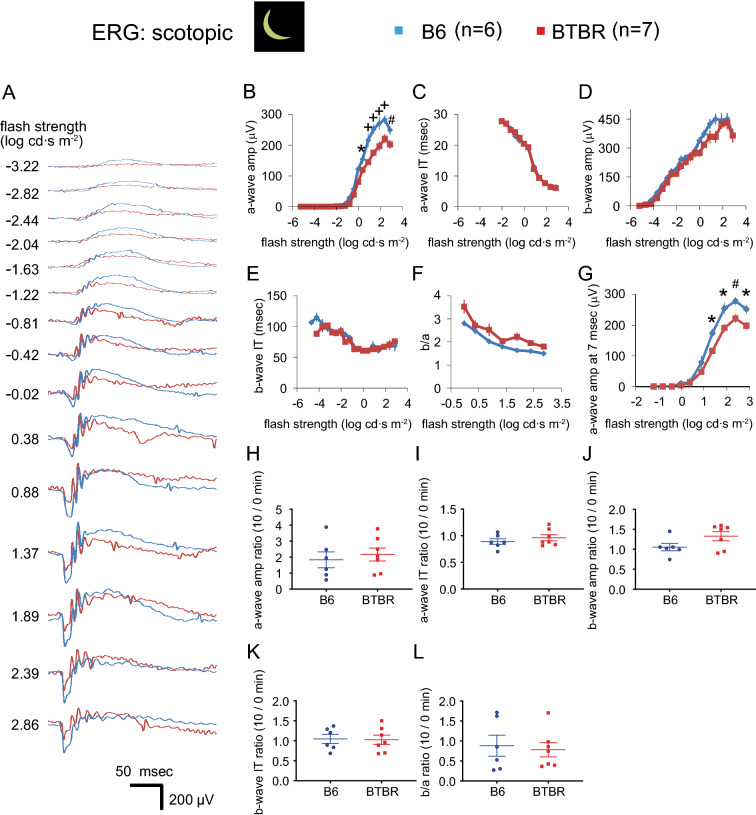
a-wave amplitudes of scotopic ERG responses were reduced in the BTBR mice. (**A**) Dark-adapted intensity responses. Examples of traces recorded at increasing flash strengths under no light background. Flash onset is at 20 ms. Bandpass is at 0.3–300 Hz. (**B**–**G**) Measurements of intensity responses are provided for a-wave amplitudes (**B**) and implicit times (**C**) as well as b-wave amplitudes (**E**) and implicit times (**E**), and for the b-wave to a-wave (b/a) amplitude ratios at 1.36 log cd∙s m^−2^ stimulus strength (**F**). a-wave amplitudes at 7 ms post-stimulus are shown in (**G**). Amplitudes were measured at 7 ms to exclude post-synaptic contribution to the a-wave. (**H–L**) The ratios of ERG responses elicited by a stimulus of 1.36 log cd∙s m^−2^, at time 0 and 10 min following transition from light (30 cd m^−2^) to dark background, are provided for the following parameters: a-wave amplitudes (**H**) and implicit times (**I**); b-wave amplitudes (**J**) and implicit times (**K**); and for b/a amplitude ratios (**L**). n = 6 B6 male mice, n = 7 BTBR male mice. For ERG intensity/response functions, data from B6 and BTBR mice were compared using two-way repeated-measures ANOVA, followed (if P < 0.05) by Sidak post-hoc pairwise comparison. In panel (**B**) and (**G**), P < 0.01 (**B**) and P < 0.05 (**G**) for two-way repeated-measures ANOVA comparing a-wave amplitudes between B6 and BTBR mice; *indicates P < 0.05, ^#^indicates P < 0.01, and ^+^indicates P < 0.0001 for post-hoc pairwise comparison. For ERG dot plots, the data were compared using the non-parametric Mann–Whitney U test. Data represent mean ± SEM. *IT* implicit time.

ERG responses were recorded to compare retinal processing between B6 and BTBR mice. As employed here, flash presentation evokes a negative-going a-wave (due to closure of cGMP-gated cationic channels in photoreceptors), followed by a positive-going b-wave (due to post-synaptic disinhibition of ON bipolar cells) onto which are superimposed oscillatory potentials (Fig. 1A). Visual stimulus processing by the rod pathway was assessed under scotopic conditions. Responses were evoked by single flash presentations at 19 increasing flash strengths, from − 5.22 to 2.86 log cd∙s m^−2^. Representative responses are shown in Fig. 2A. We measured a-wave amplitudes and implicit times (Fig. 2B,C), b-wave amplitudes and implicit times (Fig. 2D,E), and b-wave to a-wave (b/a) amplitude ratios, at 1.36 log cd∙s m^−2^ (Fig. 2F). We also measured a-wave amplitudes at 7 ms post-stimulus (Fig. 2G), to exclude post-synaptic contribution. In addition, we measured the ratios of ERG response parameters for a stimulus of 1.36 log cd∙s m^−2^, at time 0, and those at 10 min after the transition from light (30 cd m^−2^) to dark, as follows: a-wave amplitudes and implicit times (Fig. 2H,I); b-wave amplitudes and implicit times (Fig. 2J,K); and b/a amplitude ratios (Fig. 2L). Decreased a-wave amplitudes were observed in the BTBR mice (Fig. 2A,G), also when measured at 7 ms post-stimulus, further supporting a reduction in pre-synaptic photoreceptor function in BTBR versus B6 mice. Other ERG characteristics of the BTBR mice were similar to those of the B6 mice.

### Altered scotopic oscillatory potentials in the BTBR mice

**Figure 3:**
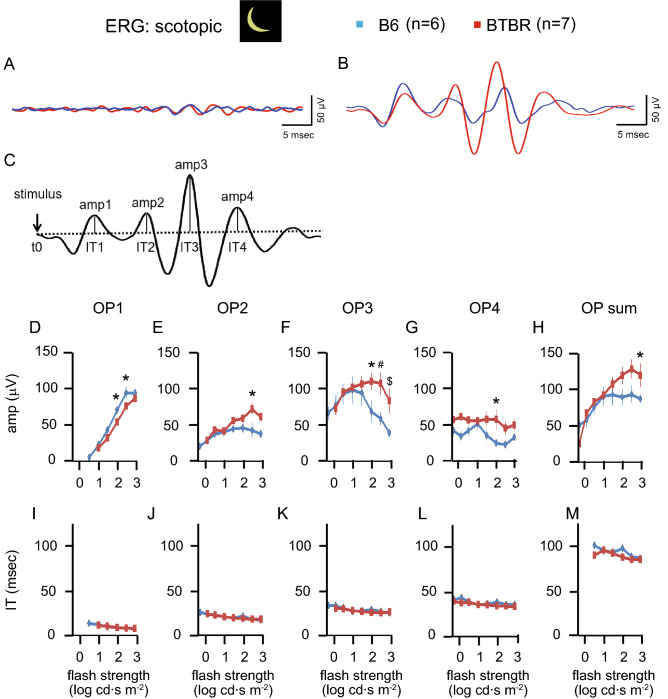
Analysis of scotopic oscillatory potentials. Panels (**A**) and (**B**) show representative traces of oscillatory potentials (OP, filtered at 75–300 Hz bandpass), elicited at stimulus strengths both below (− 2.44 log cd∙s m^−2^) and above (1.36 log cd∙s m^−2^) cone thresholds. (**C**) Four distinct OPs were quantified for their amplitudes and implicit times, as illustrated in the trace filtered with a bandpass of 75–300 Hz. Graphs show intensity/response series values of amplitudes (**D**–**H**) and implicit times (**I**–**M**), for each of the four OPs as well as for the sum of all OPs. n = 6 B6 male mice, n = 7 BTBR male mice. Comparisons were made with two-way repeated-measures ANOVA—followed by Sidak post-hoc pairwise comparison when P < 0.05: *indicates P < 0.05, ^#^indicates P < 0.01, and ^$^indicates P < 0.001. *IT* implicit time.

Representative traces of oscillatory potentials (OP), elicited at stimulus strengths below (− 2.44 log cd∙s m^−2^) and over (1.36 log cd∙s m^−2^) cone thresholds, are shown in Fig. 3A,B, respectively. Following methods reported previously^44^, four distinct OPs were quantified for their amplitudes and implicit times, as illustrated in the trace filtered with a bandpass of 75–300 Hz (Fig. 3C). Intensity/response series of amplitudes (Fig. 3D–H) and implicit times (Fig. 3I–M) were quantified for each of the four OPs, as well as for the sum of all OPs. Differences were observed in OP amplitudes, but implicit times were similar, between B6 and BTBR mice. Amplitudes were selectively reduced in OP1, contrasting with increases for the other three OPs, in the BTBR mice. All changes occurred with flash strengths in ranges that exceeded cone thresholds. These findings are in agreement with the possibility of impaired photoreceptor input, accompanied by post-photoreceptor compensation in the downstream retinal circuitry.

### BTBR mice displayed altered ERG response under photopic conditions

**Figure 4:**
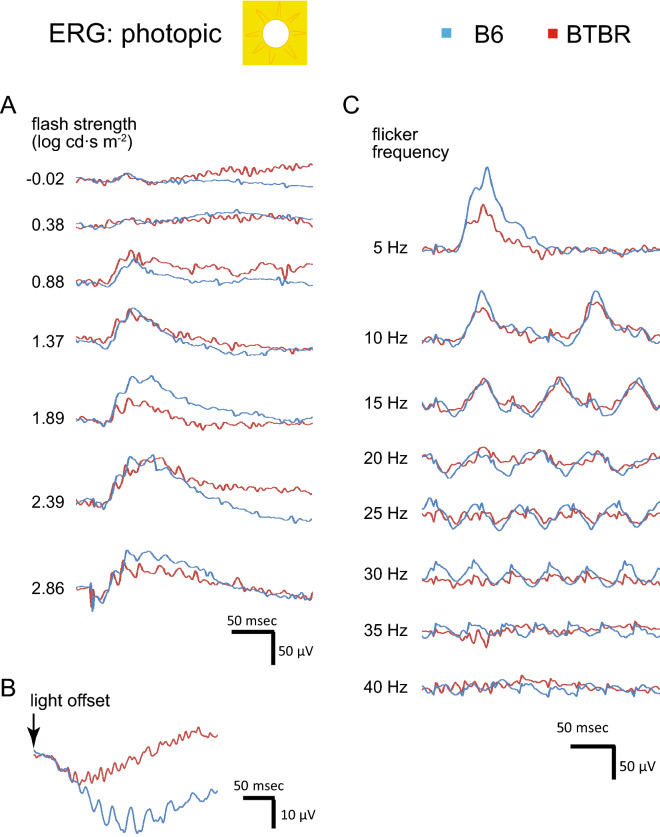
Representative traces of photopic ERG responses, comparing B6 and BTBR mice. (**A**) Photopic intensity responses. Examples of individual ERG traces recorded at increasing stimulus strengths under 30 cd m^−2^ background illumination. Flash onset is at 20 ms. (**B**) Photopic OFF responses. Examples of individual ERG traces recorded to the offset of an 800 ms square-wave light stimulus, presented at 300 cd∙s m^−2^ over a 30 cd m^−2^ background illumination. (**C**) Photopic flicker responses. Representative individual ERG traces recorded at increasing flicker frequencies under 30 cd m^−2^ background illumination. Each flash is delivered at 1.37 log cd∙s m^−2^. Bandpass is at 0.3–300 Hz for all responses.

**Figure 5:**
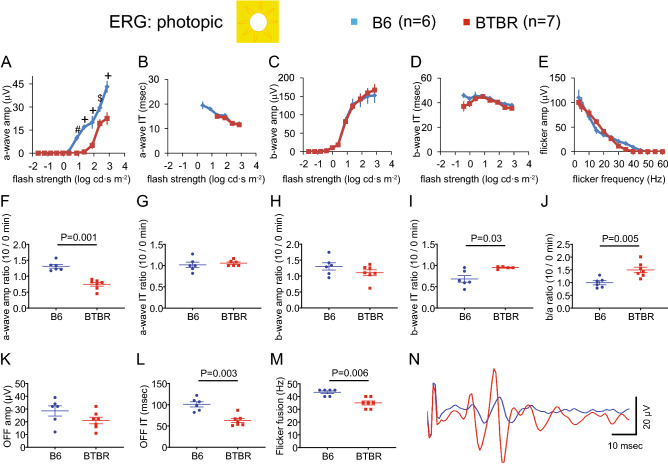
BTBR mice displayed altered photopic ERG responses. Intensity responses are provided for a-wave amplitudes (**A**) and implicit times (**B**) as well as b-wave amplitudes (**C**) and implicit times (**D**). Note: at stimulus strength ≤ 1 log cd∙s m^−2^, no a-wave response was observed in the BTBR mice. Panel **E** shows flicker response amplitudes as a function of stimulus frequency; the corresponding critical flicker fusion frequencies are provided in panel **M**. The ratios of ERG responses elicited by a stimulus of 1.36 log cd∙s m^−2^, at time 0 and 10 min following transition from dark to light (30 cd m^−2^) background, are provided for the following parameters: a-wave amplitudes (**F**) and implicit times (**G**); b-wave amplitudes (**H**) and implicit times (**I**); and for b/a amplitude ratios (**J**). Panels **K** and **L** show the OFF response amplitudes and implicit times, respectively. Finally, panel **N** shows representative traces of oscillatory potentials (filtered at 75–300 Hz bandpass). n = 6 B6 male mice, n = 7 BTBR male mice. For ERG intensity/response functions, data from B6 and BTBR mice were compared using two-way repeated-measures ANOVA, followed (if P < 0.05) by Sidak post-hoc pairwise comparison. In panel A, P < 0.001 for two-way repeated-measures ANOVA comparing a-wave amplitude between B6 and BTBR mice; ^#^indicates P < 0.01, ^$^indicates P < 0.001, and ^+^indicates P < 0.0001 for post-hoc pairwise comparison. For ERG dot plots, the data were compared using the non-parametric Mann–Whitney U test. Data represent mean ± SEM. *IT* implicit time.

Visual signal processing in the cone pathway was assessed under photopic conditions. Ten minutes after the transition from dark- to light-adapted (30 cd m^−2^ white light background), responses were recorded at 11 increasing flash strengths ranging from − 1.6 to 2.9 log cd∙s m^−2^. Examples of responses are shown in Fig. 4A. We also studied photopic OFF responses to the offset of an 800 ms square wave light stimulus presented at 300 cd∙s m^−2^ (Fig. 4B), and flicker responses to increasing flicker frequencies delivered at 1.37 log cd∙s m^−2^ (Fig. 4C). Under these conditions, a-wave amplitudes, as a function of flash strength, were significantly smaller and had higher threshold in the BTBR mice (Fig. 5A); in contrast, a-wave implicit times (Fig. 5B), as well as b-wave amplitudes and implicit times (Fig. 5C,D), were similar between the two strains. Flicker amplitudes as a function of stimulus frequency were similar in the two strains (Fig. 5E), but the critical flicker fusion frequency (the highest temporal frequency at which a stimulus can be distinguished as flickering rather than continuous) was significantly lower in the BTBR mice (Fig. 5M). In addition, the ratios of ERG responses elicited by a stimulus of 1.36 log cd∙s m^−2^, at time 0 compared to 10 min following transition from dark to light (30 cd m^−2^), were measured for the following parameters: a-wave amplitudes and implicit times (Fig. 5F,G); b-wave amplitudes and implicit times (Fig. 5H,I); and b/a amplitude ratios (Fig. 5J). The BTBR mice displayed a significantly lower ratio of a-wave amplitudes (Fig. 5F) and a higher ratio of b/a amplitude ratios (Fig. 5J), as well as a higher ratio of b-wave implicit times (Fig. 5I) than B6 mice. The OFF response amplitudes and implicit times are shown in Fig. 5K,L, respectively; BTBR mice exhibited significantly shorter implicit time of the OFF response (Fig. 5L). Finally, representative traces of oscillatory potentials (filtered at 75–300 Hz bandpass) are shown in Fig. 5N.

### Increased spatial acuity in the BTBR mice under both scotopic and photopic light conditions

Retinal afferents project to different visually-responsive areas of the brain^45^. Any developmental discrepancies occurring along these pathways have the potential of impacting the processing of visual stimuli. To compare visual function between B6 and BTBR mice and probe for potential further contributions of post-retinal processing, the optokinetic response (OKR)—an innate visuo-motor reflex—was used to measure spatial contrast sensitivity and spatial acuity under photopic and scotopic conditions. Spatial acuity, and contrast sensitivity with its basis in spatial frequency channels, are ideal for examining the integrity of lower-level (retinal + subcortical) visual function.

**Figure 6:**
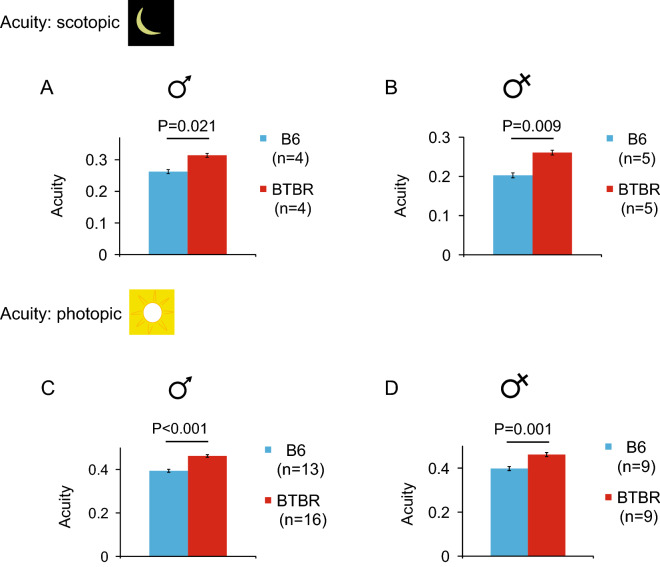
Increased spatial acuity in the BTBR mice under both light conditions. Visual acuity measured by threshold spatial frequency in male and female B6 and BTBR mice, under scotopic conditions (**A & B**) and photopic conditions (**C & D**). Acuity was measured at a fixed temporal frequency of 0.40 Hz and 100% spatial contrast. To compare acuity of B6 and BTBR mice under each condition, the Mann–Whitney U test was used. Data represent mean ± SEM.

Under scotopic conditions (mean luminance = − 4.32 log cd m^−2^), male BTBR mice displayed significantly higher spatial acuity (0.314 ± 0.005 cyc/deg) than male B6 controls (0.262 ± 0.006 cyc/deg; P = 0.021) (Fig. 6A). Similarly, female BTBR mice demonstrated significantly higher spatial acuity (0.260 ± 0.010 cyc/deg) than female B6 controls (0.200 ± 0.006 cyc/deg; P = 0.009) (Fig. 6B).

Under photopic conditions (mean luminance = 1.98 log cd m^−2^), as under scotopic conditions, male BTBR mice achieved significantly higher spatial acuity (0.462 ± 0.006 cyc/deg) than male B6 controls (0.394 ± 0.007 cyc/deg; P < 0.001) (Fig. 6C). Similarly, female BTBR mice achieved higher spatial acuity (0.461 ± 0.009 cyc/deg) than female B6 controls (0.397 ± 0.009; p = 0.001) (Fig. 6D).

### Increased contrast sensitivity at high spatial frequencies in the BTBR mice

**Figure 7:**
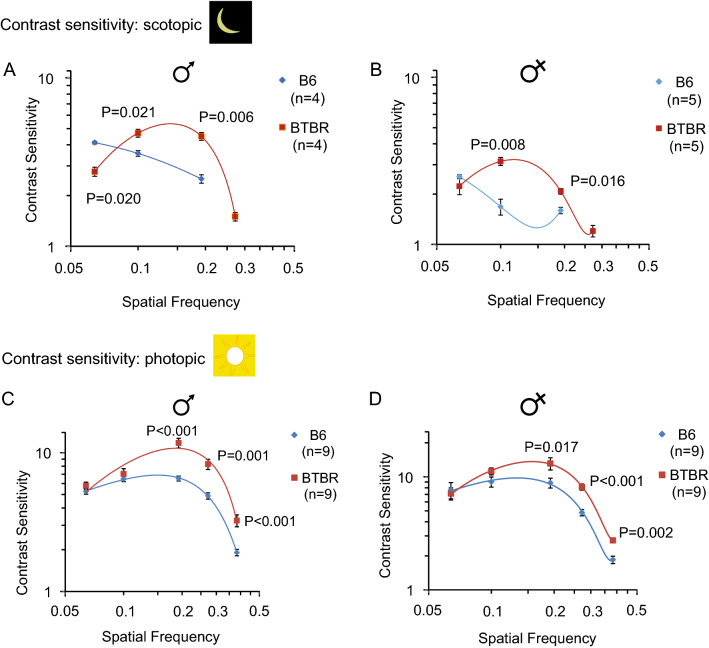
Increased contrast sensitivity at high spatial frequencies in the BTBR mice. Contrast sensitivity measured at the following spatial frequencies: 0.064, 0.100, 0.192, 0.272 and 0.383 cyc/deg, in male (**A**) and female (**B**) B6 and BTBR mice under scotopic conditions. No response was elicited at 0.272 cyc/deg from the B6 controls of either sex, and no response was elicited at 0.383 cyc/deg from either strain of mice. Similarly, contrast sensitivity measured in male (**C**) and female (**D**) B6 and BTBR mice under photopic conditions. To compare contrast sensitivity of B6 and BTBR mice at each spatial frequency, under scotopic or photopic conditions, the Mann–Whitney U test was used. Data represent mean ± SEM.

Contrast sensitivity was tested at five spatial frequencies: 0.064, 0.100, 0.192, 0.272 and 0.383 cyc/deg. Under scotopic conditions, contrast sensitivity was significantly greater at 0.100 and 0.192 cyc/deg, but significantly less at 0.064 cyc/deg, in male BTBR mice than in male B6 controls (Fig. 7A). In the case of female mice, spatial contrast sensitivity was significantly greater at 0.100 and 0.192 cyc/deg in the BTBR mice (Fig. 7B), as in the male mice; however, no significant difference was observed at 0.064 cyc/deg. For both males and females, OKR was not elicited in the B6 group at 0.272 cyc/deg; while at 0.383 cyc/deg, no mouse from either strain exhibited visual tracking. It was likely that most of the mice were unable to detect the drifting gratings at this spatial frequency under these scotopic conditions. Note that the curve-fitting for data from the BTBR mice resulted an inverted U, as reported before^34^; however, for the B6 group, contrast sensitivity was maximal at the lowest spatial frequency tested (0.064 cyc/deg), so that the standard inverted U-shape of the curve was not observed. Compared to the results under photopic conditions shown below, the results under scotopic conditions were consistent with those obtained in previous studies, which found a leftward shift of peak contrast sensitivity with reduced intensity of the visual stimulus^34^.

Under photopic conditions, contrast sensitivity at spatial frequencies of 0.192, 0.272 and 0.383 cyc/deg was higher in the BTBR mice than in B6 controls, in both males (Fig. 7C) and females (Fig. 7D). No significant differences were observed between contrast sensitivity functions of the BTBR and the B6 mice, of either sex, at the lowest spatial frequencies tested (0.064 and 0.100 cyc/deg).

## Discussion

### Altered retinal function in the BTBR mice probed by ERG

Visual processing starts in photoreceptors with the photo-isomerization of 11-cis-retinal (derived from Vitamin A and covalently bound to opsin proteins) into all-trans-retinal, which activates the G-protein, transducin^46,47^. Transducin in turn activates a phosphodiesterase that hydrolyzes cGMP into GMP, closing cGMP-gated cationic channels and thereby hyperpolarizing photoreceptors, which causes a drop in presynaptic glutamate exocytosis^48^. Illumination thus modulates the activity of post-synaptic targets—bipolar cells and horizontal cells—and through them, inner-retinal interneurons (amacrine cells), and retinal ganglion cells, which provide the only retinal output to the brain.

We observed complex differences between the ERGs of the BTBR and B6 mice. In the BTBR mice, a-wave amplitudes were reduced under both scotopic (Fig. 2) and photopic (Figs. 4, 5) conditions, indicating decreased responses to light by rod and cone photoreceptors. Upon shifting from scotopic to photopic conditions, BTBR mice failed to show the increase in a-wave amplitude and reduction in b-wave implicit time observed in the B6 mice (Fig. 5F,I), suggesting a deficit in light adaptation in the cone pathway. Critical flicker fusion frequency was also lower in BTBR than in B6 mice, indicating poorer temporal resolution in the retinal circuitry responsible for the flicker response. A possible mechanism underlying these changes is alteration of calcium homeostasis in the outer retina^49^, which warrants further investigation. Oscillatory potentials were different between the two strains of mice (Fig. 3). While the neural mechanisms of oscillatory potentials are not completely understood, it is generally believed that they are generated by neural circuits in the retina, in particular those involving amacrine cells, and that early and later OPs are associated with the activity of photoreceptors/bipolar cells and amacrine/ganglion cells in the retina, respectively^50,51^. Our results from BTBR mice revealed selectively reduced amplitudes of OP1, while the subsequent three OPs had increased amplitudes; all differences systematically occurred at flash strength ranges exceeding the cone thresholds. These findings suggest impairment of photoreceptor input, accompanied by post-photoreceptor compensation in the retinal circuitry. A limitation of the current study is that reliance upon the ERG for electrophysiological assessment precluded the identification of possible strain-dependent differences in responsiveness and functional organization of retinal ganglion cells (RGCs), since it is generally believed that the activity of these cells does not contribute to the ERG. We were able to assess differences in RGC activity only in a limited way, that of the unique subset of directionally-selective ganglion cells that drive the OKR.

Retinal defects have been reported in previous studies using other models of ASD. In a study of *En2* knockout mice, amplitudes of both a-wave and b-wave in scotopic ERG response were reduced, while the photopic ERG was preserved^19^. In another study of *Fmr1* knockout mice, both a-wave and b-wave amplitudes in scotopic ERG were decreased^18^. Interestingly, a recent article reported that in the valproic acid induced mouse model of ASD, a-wave amplitudes were smaller yet b-wave amplitudes were normal in scotopic ERG^20^, similar to what we observed here. Thus, it appears that a decrease in a-wave amplitude is shared by idiopathic and syndromic mouse models of ASD.

Studies using the ERG to investigate retinal processing in patients with ASD have been very limited. Our observations of altered retinal processing, particularly in the cone pathway, are reminiscent of those that demonstrate altered ERG responses in people with ASD^52–54^—especially changes in the b-wave, suggesting altered transmission from cones to ON-bipolar cells.

### Enhanced visual function at only higher spatial frequencies in the BTBR mice observed under both scotopic and photopic conditions

The characteristics of optokinetic spatial acuity and contrast sensitivity in the B6 mice are consistent with those reported previously, in that visual acuity and contrast sensitivities were lower in dark-adapted than in light-adapted conditions. Under scotopic conditions, Umino et al. found that at luminance =  − 4.5 log cd m^−2^ and speed = 0.8 Hz, contrast sensitivity reached ~ 5 at 0.128 cyc/deg^34^. In addition, Cowan et al., and van der Heijden et al. found that at luminance =  − 2.3 log cd m^−2^ and speed = 2 Hz, contrast sensitivity reached ~ 3.5 at 0.08 cyc/deg^55,56^. In the present study, we found that at luminance =  − 4.32 log cd m^−2^ and speed = 0.8 Hz, contrast sensitivity reached 4.12 at 0.064 cyc/deg. Therefore, our results are in the same range as previously published data. In addition to luminance level and stimulus parameters, there may be other conditions that differed in these studies, including that, after breeding for a few generations, each laboratory has a unique sub-colony of the original strain of mice due to spontaneous mutations (Jackson Laboratory), and that different environment such as season and humidity may affect the visual behaviour. Thus, it is not surprising that results reported by different groups have a spread. Importantly, we tested the BTBR and B6 mice in parallel, minimizing any differences that might otherwise have been introduced by experimental conditions when comparing the two.

We found that spatial acuity, and contrast sensitivity at higher spatial frequencies, were greater in BTBR than in B6 mice, under both photopic and scotopic conditions; that is, processing of visual information of relatively high spatial frequency is enhanced in the BTBR mice. A similar phenotype has been reported among individuals with ASD, as described in more detail below.

In one human study, contrast sensitivities for both luminance and texture-defined vertically-oriented sine-wave gratings were measured across a range of spatial frequencies; the results showed that autistic participants were more sensitive to luminance-defined, high spatial frequency gratings (8 cpd)^57^. Another study found that many aspects of facial image processing were deficient in children with ASD, but these children showed better performance when using information of high (i.e., local facial features) rather than low (i.e., global configuration of faces) spatial frequency for matching faces^58^. Furthermore, by measuring visually evoked potentials, another study demonstrated that adults with ASD exhibited enhanced fine-form (local high spatial frequency) processing, but impaired gestalt face processing due to deficient integration of various local high spatial frequency information in area V4^59^. Together, these studies suggest that locally-biased perception in ASD originates, at least partially, from alterations in early spatial response mechanisms favouring detailed spatial information processing, which may lead to atypical social recognition and interaction.

However, these findings are not universal, and mixed results from the literature have given rise to much debate. For example, while some studies report ‘super vision’ in patients with ASD, later studies have generally supported the notion that visual acuity is normal in this population^60–68^. In addition, other studies have found no difference in contrast sensitivity between ASD and control groups^61,69^. The variety of visual phenotypes reported in the literature may be due to the fact that ASD is a spectrum disorder, in which the severity and presentations of the symptoms vary over a very large range. Additionally, there have been many challenges in measuring visual function (including spatial acuity and contrast sensitivity) in this population, especially in children, because of wide variations in testing methods, sample sizes, selection of high‐functioning participants, cooperativeness during visual testing, and social and communication difficulties^12–14^.

Taken together, studies on ASD-related atypical vision have reported mixed results, and further investigations will be necessary to understand the nature of these observed variations, potentially with the help of animal models of ASD. Our results using the BTBR mice are similar to those that have been reported in a subset of patients with ASD, supporting the hypothesis that differential sensitivity to low versus high spatial frequencies might play a causal role in the anomalies of detail-oriented visual perception in this population. Further studies to elucidate the relationships between visual function and the robust social and repetitive behaviour phenotype of the BTBR mice are warranted, in the context of continued efforts to explore the relationship between visual perception and social development in people with ASD. In addition, the cause of the superior visual acuity and contrast sensitivity in the BTBR mice remains unknown, and further experiments are needed. The results of our ERG and OKR tests suggest that in the BTBR mice, while enhanced visual behaviors under both photopic and scotopic conditions might be due to alterations in visual processing common to both rod and cone pathways, these mechanisms are probably downstream of photoreceptor function; they might include inner-retinal processing at the level of amacrine and ganglion cells, as well as post-retinal processing in the brain, with possible alteration in either the intrinsic properties of these neurons or the synaptic connections between different cell types or processing stages. For example, our analysis of oscillatory potentials suggests that changes in post-photoreceptor retinal circuitry might represent a compensatory event to reduced photoreceptor input, and contribute, at least partially, to our observation of enhanced visual functions measured behaviourally in the BTBR mice. Notably, disrupted visual circuitry in the brain has been reported in this mouse model^29^; altered anatomical shape and impaired synaptic pruning have been observed in the dorsal lateral geniculate nucleus (dLGN), a key component in the visual pathway, although not directly related to the processing of OKR-associated stimuli. The dLGN is located close to the medial terminal nucleus, which is part of the accessory optic system mediating the OKR^38–43^. It is possible that local disruptions caused by LGN deformation contribute to the anomalous OKR phenotype observed here. In addition, recent studies have revealed changes in both structural and functional connectivity of the primary visual cortex in the BTBR mice^70,71^, providing further evidence that visual pathways and central visual processing are abnormal in this model. Finally, many investigations in the BTBR mice have revealed other differences in genetic and epigenetic regulation, neurotransmission, and structural and functional connectivity in the brain, as well as in the immune system^21–25,72,73^. Given that many of these differences are similar to those found in individuals with ASD^3,22,24^, they might contribute to the atypical visual processing described here.

Sex differences in visual function have not been thoroughly investigated; previous studies reported either no sex difference in several inbred strains of mice when performing visual detection or pattern discrimination tasks using a two-alternative swim task^74^, or lower gain of eye movement as part of the gaze-stabilization reflex in female B6 mice^75^. Here, we also compared the B6 males with B6 females, and BTBR males with BTBR females in terms of both acuity and contrast sensitivity, under both scotopic and photopic conditions. Our results showed that under the scotopic condition, male mice displayed greater acuity and contrast sensitivity than female mice, in both B6 and BTBR strains. However, under the photopic condition, female B6 mice achieved greater contrast sensitivity than male B6 mice at 0.1 and 0.192 cycles per degree, and female BTBR mice achieved greater contrast sensitivity than male BTBR mice at 0.1 cycles per degree. Thus, the difference in visual function between male and female mice is complex, depending on the light condition, the parameter being tested, and the strain of mice. The differences in observations from different studies could be due to different testing paradigms and lighting conditions. Interestingly, it has been reported that human males outperform females in some visual tasks^76,77^. The difference in visual function between male and female mice is intriguing, and it was important to investigate visual function in female mouse models of ASD—not only because male and female physiologies are different, but also because both men and women present with autism. Although the prevalence of ASD is higher in human males than in females, it should not be assumed that results from males are generalizable to the entire patient population.

Interestingly, a previous study on the effects of aging on the BTBR mice, using unbiased proteomic analysis of hippocampal and cortical tissue of 15-month-old mice, revealed significant changes in the levels of BDNF and multiple synaptic proteins^78^. Although the current study was performed on 8- to 14-week-old mice, it is possible that the aging of visual functions might be different between the BTBR and B6 mice; this would be interesting to explore in future studies.

One of the limitations of the current study is that the visual functions measured by visual reflex (OKR) in mice are not necessarily the same as the parameters measured in humans. As mentioned earlier, the visual reflex is driven by visual information delivered by a subset of direction-selective ganglion cells, to the central targets of the accessory optic system, subject to some modifications by input from other brain regions^38–41^. In contrast, in cognitive testing of human visual performance, visual processing is dominated by activity in higher centers including the lateral geniculate nucleus and visual cortex. Thus, while we used the visual reflex as a proxy for human visual function with indirect translation, the restricted functions of the direction-selective retinal ganglion cells and the accessory optic system might be expected to reveal visual processing somewhat different from those due to other ganglion cells that project to the main visual system in the brain.

As mentioned earlier, studies on visual behavior in animal models of ASD have been limited^15–19^. One of those studies examined a mouse model of MECP2 duplication syndrome, which overlaps phenotypically with ASD. In vivo extracellular recordings in MECP2 transgenic mice showed that neurons in V1 preferred higher spatial frequencies than the corresponding neurons in wildtype controls. Using a two-alternative forced-choice visual detection task—which tests preferentially central (cortical) over peripheral (retinal) visual functions—the authors also found that visual discrimination of spatial frequencies at 0.24 and 0.35 cyc/deg, and contrasts ranging from 40 to 100%, was significantly better in transgenic than in wildtype mice^15^. Additionally, in the *En2* knockout mice, it has been shown that baseline binocularity and plasticity are altered^17^. Moreover, a separate study demonstrated that *Foxg1* haploinsufficiency caused impairment of visual cortical function in mouse and human^16^. Similar cortical recordings and behavioral tests could be applied to the BTBR mice as well, to further our understanding of the visual phenotype reported here. In addition, the anatomical and biochemical characterization of the retina and visual cortex, used in previous studies, provides a useful guide^15–20^, which could be followed in subsequent studies to investigate possible structural or biochemical rearrangements underlying the altered visual function in BTBR mice.

It has been reported that the B6 mice displayed greater contrast sensitivity than the 129/SvEv strain^79^, yet in the current study the BTBR mice performed even better than the B6 mice. In addition, a study that measured visual function using two-alternative swim task in 14 strains of mice revealed significant differences in visual performance among strains^74^. It is possible that visual function, as measured by reflex or cognitive behaviors in different strains of mice, spreads over a broad spectrum, and that BTBR mice are on the high-function end of that spectrum.

### Investigating visual functions in ASD

Neuroimaging studies on individuals with ASD can help to identify potential mechanisms resulting in atypical visual function. Interestingly, brainstem hypoplasia has been reported in all cases of autism studied to date, regardless of age^80^. As discussed previously, the OKR response is mediated by the accessory optic system, which relies on midbrain (a part of the brainstem) nuclei to relay information to the motor system. In the BTBR mice, while deformation in the dLGN has been reported^29^, it has yet to be investigated whether structural abnormalities are also present along the accessory optic system.

Studies on interventions for alleviating visual problems in individuals with ASD have been very limited^81,82^, and results have been mixed. Understanding alterations in vision could help in discovering ways to meet the needs of individuals with autism, and thus to mitigate the symptoms of the disorder. Investigating visual function in animal models of ASD may offer new insights into the relationships between altered visual function and autism-like behavioral phenotypes.

As there is currently no cure for ASD, individuals often cope with this neurological disorder for life^1^. Not only are symptom-management therapies very expensive, but also—because of the heterogeneous nature of the disorder—there is no guarantee that a given individual will benefit from a specific treatment regimen. More often than not, individuals with ASD face debilitating social barriers, which prevent them from achieving an adequate quality of life. Therefore, more insight into the underlying connections between sensory functions (including visual processing) and behaviour may prompt the discovery of novel and more cost-effective means of managing ASD.

## Conclusions

In summary, the present studies of visual function in the BTBR mouse model of ASD have revealed altered intraretinal processing, mostly related to the cone pathway, as well as enhanced visual discrimination of finer spatial details and improved contrast sensitivity at higher spatial frequencies, under both cone- and rod-dominant conditions. These results are similar to findings reported by others in a subset of patients with ASD, supporting the hypothesis that differential sensitivities to low versus high spatial frequencies may play a role in detail-oriented visual perception in this population. Taken together, these results indicate that BTBR mouse is a promising animal model for understanding further the visual phenotype associated with ASD.

## Data Availability

Data are available upon request.
